# DNA Methylation Profile Distinguishes Clear Cell Sarcoma of the Kidney from Other Pediatric Renal Tumors

**DOI:** 10.1371/journal.pone.0062233

**Published:** 2013-04-26

**Authors:** Hitomi Ueno, Hajime Okita, Shingo Akimoto, Kenichiro Kobayashi, Kazuhiko Nakabayashi, Kenichiro Hata, Junichiro Fujimoto, Jun-ichi Hata, Masahiro Fukuzawa, Nobutaka Kiyokawa

**Affiliations:** 1 Department of Pediatric Hematology and Oncology Research, National Research Institute for Child Health and Development, Setagaya-ku, Tokyo, Japan; 2 Department of Maternal-Fetal Biology, National Research Institute for Child Health and Development, Setagaya-ku, Tokyo, Japan; 3 Director of Clinical Research Center, National Center for Child Health and Development, Setagaya-ku, Tokyo, Japan; 4 College of Human Science, Tokiwa University, Mito, Ibaraki, Japan; 5 President of Osaka Medical Center and Research Institute for Maternal and Child Health, Osaka Medical Center and Research Institute for Maternal and Child Health, Izumi, Osaka, Japan; The Chinese University of Hong Kong, Hong Kong

## Abstract

A number of specific, distinct neoplastic entities occur in the pediatric kidney, including Wilms’ tumor, clear cell sarcoma of the kidney (CCSK), congenital mesoblastic nephroma (CMN), rhabdoid tumor of the kidney (RTK), and the Ewing’s sarcoma family of tumors (ESFT). By employing DNA methylation profiling using Illumina Infinium HumanMethylation27, we analyzed the epigenetic characteristics of the sarcomas including CCSK, RTK, and ESFT in comparison with those of the non-neoplastic kidney (NK), and these tumors exhibited distinct DNA methylation profiles in a tumor-type-specific manner. CCSK is the most frequently hypermethylated, but least frequently hypomethylated, at CpG sites among these sarcomas, and exhibited 490 hypermethylated and 46 hypomethylated CpG sites in compared with NK. We further validated the results by MassARRAY, and revealed that a combination of four genes was sufficient for the DNA methylation profile-based differentiation of these tumors by clustering analysis. Furthermore, *THBS1* CpG sites were found to be specifically hypermethylated in CCSK and, thus, the DNA methylation status of these *THBS1* sites alone was sufficient for the distinction of CCSK from other pediatric renal tumors, including Wilms’ tumor and CMN. Moreover, combined bisulfite restriction analysis could be applied for the detection of hypermethylation of a *THBS1* CpG site. Besides the biological significance in the pathogenesis, the DNA methylation profile should be useful for the differential diagnosis of pediatric renal tumors.

## Introduction

In the pediatric population, the types of renal tumor are entirely different from those occurring in adults. It is estimated that 85% of pediatric renal malignancies comprise nephroblastoma, 5% congenital mesoblastic nephroma (CMN), 4% clear cell sarcoma of the kidney (CCSK), and 2% rhabdoid tumor of the kidney (RTK) [1], and these 4 major entities account for 96% of the total. The remaining 4% tend to occur in older children and include miscellaneous tumors, such as the Ewing’s sarcoma family of tumors (ESFT). Nephroblastoma is malignant but still a relatively favorable tumor prognostically, being derived from nephrogenic blastemal cells that can show divergent differentiation. CMN is a kind of fibroblastic sarcoma of infancy and characterized by a specific chromosomal translocation, t(12;15)(p13;q25), which results in the fusion of *ETV6* and *NTRK3* genes [2]. On the other hand, CCSK is a relatively unfavorable tumor prognostically, being composed of clear mesenchymal cells with a characteristic vascular pattern [3]. RTK is a highly aggressive tumor occurring in young children, has a dismal outcome, and is characterized by pathological rhabdoid features and molecular biallelic inactivation of the *SMARCB1* (*hSNF5/INI1*) gene [4]–[6].

Since pediatric renal tumors are diverse neoplastic entities, as described above, and require different therapeutic strategies, rapid and accurate diagnosis is crucial for adequate treatment. However, all those tumors are composed of small-to-medium-sized, round, oval, or spindle-shaped undifferentiated or immature cells, and often deceptively mimic each other, making the diagnosis difficult [7]. In RTK and CMN, molecular markers, i.e., loss of SMARCB1 expression and *ETV6-NTRK3* fusion, respectively, are useful for an ancillary diagnosis, whereas the diagnosis of nephroblastoma and CCSK is exclusively based on histologic features. Although numerous studies have been done, immunohistochemical features or recurrent genetic changes that can reliably distinguish CCSKs from other pediatric renal tumors have not identified [3], [8]. Therefore, the identification of molecular signatures that can distinguish CCSK from other renal tumors should be useful and provide diagnostic confidence and accuracy.

Alterations of DNA methylation have been well documented as an important peculiarity of cancer cells [9], [10], and two patterns of DNA-methylation changes have been observed in cancer [11], [12]. One is a global hypomethylation associated with increased chromosomal instability, the reactivation of transposable elements, and loss of imprinting. The other is hypermethylation of CpG islands located in promoter regions of tumor suppressor genes that has conventionally been associated with transcriptional silencing in cancer. These aberrant DNA methylations are thought to be closely related to the development of cancer. Therefore, the identification of specific DNA methylation markers would be helpful for understanding the pathogenetic mechanism as well as for developing new therapeutic strategies. In Wilms’ tumor, hypermethylation of *HACE1*, *RASSF1A* and *SIM1* and hypomethylation of *GRIPR* were reported [13]–[16], whereas the DNA methylation analysis in pediatric renal sarcomas including RTK, CCSK has not been reported yet.

In an attempt to investigate the characteristics of DNA methylation of pediatric sarcomas including CCSK, RTK, and ESFT, we performed DNA methylation analysis using Illumina Infinium HumanMethylation27. In this paper, we demonstrated that each sarcoma had a distinct DNA methylation profile and could be classified by the methylation pattern of a set of specific genes. We further proposed a convenient assay for the differential diagnosis of CCSK from other pediatric renal tumor.

## Materials and Methods

### Ethics Statement

This study was approved by the ethics committee/IRB at the National Center for Child Health and Development, and written informed consent was obtained from parents for samples from JWiTS. Since written informed consent was not obtained in a subset of samples collected before 2001, the identifying information for them was removed before analysis, in accordance with the Ethical Guideline for Clinical Research enacted by the Japanese Government. The ethics committee/IRB approved the waiver of written informed consent for latter samples.

### Clinical Materials

Clinical specimens from pediatric patients, including 6 each with RTK and Ewing’s sarcoma, 21 with CCSK, 9 with CMN, and 41 with Wilms’ tumor, used in this study were selected from the files of specimens collected in our laboratory between 1985 and 2001, and the JWiTS (Japan Wilms Tumor Study). In each case, the pathological diagnosis was confirmed by J.H. and H.O. based on morphological observations and the immunophenotypic characteristics. Three non-neoplastic kidney (NK) tissues were obtained from the non-tumorous part of the above specimens.

### Sample Preparation

Each disease tissue was evaluated by preparing frozen section and the neoplastic cells accounted for more than 80% of viable cells in each sample. The pathologic images of the disease tissues were presented in Figure S1. Genomic DNA was extracted from above evaluated fresh-frozen tissues using the illustra tissue & cells genomicPrep Mini Spin kit (GE Healthcare Bio-Sciences UK Ltd, Chalfont, UK) according to the manufacturer’s instructions. The quantity of DNA was assessed by Quant-iT Pico-Green dsDNA Reagent and Kits (Life Technologies Corporation, Carlsbad, CA, USA) and the quality was assessed by agarose gel electrophoresis.

### Bisulfite Conversion

Starting from 1 µg of genomic DNA, bisulfite conversion of genomic DNA was performed using the Epitect Bisulfite kit (QIAGEN Inc., Valencia, CA, USA) and EZ DNA Methylation Kit (Zymo Research, Irvine, CA, USA) for the Illumina Infinium Methylation Assay and SEQUENOM MassARRAY, respectively, according to the manufacturer’s protocol.

### Infinium Methylation Assay

Illumina Infinium HumanMethylation27 (Illumina, Inc., San Diego, CA, USA), containing the 27,578 CpG sites, spanning 14,495 genes, was used for methylation analysis. The bisulfite converted DNA was processed on the chip according to the Illumina protocol. The BeadChips were scanned using iScan system. Our data of Infinium assay have been deposited on Gene Expression Omnibus (GEO) database at the National Center for Biotechnology Information (NCBI, http://www.ncbi.nlm.nih.gov/geo/, series accession number GSE44847). The β-value and the detection p-value for each locus were calculated using GenomeStudio v2010.1. β-value, a ratio of methylated probe signal intensity to sum of methylated and unmethylated probe signal intensities, was used to estimate the methylation level of the target locus. Detection p-value is computed from the background model characterizing the chance that the target sequence signal was distinguishable from negative controls. The obtained data were filtered by exclusion of the probes with a detection p-value>0.05 from all probes and SD>0.2 within each entity. The numbers of the detected CpG sites for each sample and filtering process were shown in Table S1. As a result, 23,700 probes (13,385 genes) remained.

### EPITYPER Assay (MassARRAY)

The SEQUENOM EpiTYPER assay was performed according to the protocol recommended by the manufacturer. Using the Complete PCR Reagent Set (SEQUENOM Inc., San Diego, CA, USA), target regions were amplified from bisulfite-converted DNAs using the primer pairs containing a T7-promoter tag to allow further *in vitro* transcription. The primers used in this study (Table 1) were designed by EpiDesigner (SEQUENOM). The cycle conditions used were: 95°c for 4 min, 45 cycles of 95°C for 20 s, 56°C (65°C for *CREG1*) for 30 s. and, 95°C for 1 min, and 72°C for 3 min. The PCR products were confirmed by agarose gel electrophoresis. After the dephosphorylation of unincorporated dNTPs by shrimp alkaline phosphatase (SAP) (SEQUENOM), transcription and digestion were performed simultaneously at 37°C for 3 h by RNase A and T7 polymerase (SEQUENOM). The cleavage reactants were purified with CLEAN resin (SEQUENOME) and dispensed onto silicon chips preloaded with matrix (SpectroCHIPS, SEQUENOM). Mass spectra were collected using a MassARRAY mass spectrometer (Bruker-Sequenom) and analyzed using proprietary peak picking and signal-to-noise calculations (Sequenom Epityper v1.0.5). In MassARRAY analysis, initially, quality control (QC) was performed in each CpG site.

**Table 1:** Primers used for MassARRAY.

Primer name	5' - 3' sequence
ADRA1D_F	aggaagagagTGGTAGGTAATTTGTTTGTTATTTTTTT
ADRA1D_R	cagtaatacgactcactatagggagaaggctCTTCCAACCCAACAAAAACCCTA
ALDOC_F	aggaagagagTTGAATTTGGGTATTTTGAAGATGT
ALDOC_R	cagtaatacgactcactatagggagaaggctCAAATAAAACTACAACCCTAACTCCC
CREG1_F	aggaagagagGTGAGTAATTTGTAGGTGAGTTGGG
CREG1_R	cagtaatacgactcactatagggagaaggctCCACTACACTCCAACCTAAACCA
MGMT_F	aggaagagagTGAGATTGTTAGAGTGTGTTTTTGG
MGMT_R	cagtaatacgactcactatagggagaaggctTCCACTCAAACCAACTAAATTACCTA
PKN1_F	aggaagagagGGTTTTTTTTGGAGAATTAGAAGGG
PKN1_R	cagtaatacgactcactatagggagaaggctCCAACCACCATACAAAAAAATAAAA
PTEN_F	aggaagagagGGGGTTGTAAATAGATTTGATAGGTT
PTEN_R	cagtaatacgactcactatagggagaaggctAAAAAAAATCCCCAAACTAATACCA
THBS1_F	aggaagagagGGAGAGAGGAGTTTAGATTGGTTTT
THBS1_R	cagtaatacgactcactatagggagaaggctACCTTACCCTAAAAAATCCTCCAAC
VHL_F	aggaagagagTTTTGGGGAGATTGATAGATGTAAA
VHL_R	cagtaatacgactcactatagggagaaggctAACCACTTAACCCCAAATAACAAAT

F, forward; R, reverse. Lower case letters indicate tag sequences.

### Combined Bisulfite Restriction Analysis (COBRA)

Bisulfite PCR products of *THBS1* produced as described above were digested with the methylation-sensitive restriction enzyme HpyCH4IV (5′-ACGT-3′) (New England Biolabs, NEB, Ipswich, MA, USA) for 12 h at 37°C The digested DNA was separated on 2% agarose gels in 0.5× TBE buffer, stained with ethidium bromide, and visualized on a UV transilluminator. As a control of HpyCH4IV digestion, 0, 50, and 100% methylated DNA were used.

### Statistical Analysis

Two-way hierarchical cluster analyses of Infinium assay and MassARRAY were performed using hclust in the R clustering package with Euclidean metric and complete linkage for statistical computing.

## Results

DNA methylation profiling of 3 each of CCSK, RTK, ESFT and NK, 12 samples in total, were performed using Infinium HumanMethylation27. First, we analyzed the general methylation status of each tumor group by defining hyper- and hypomethylated CpG sites in each tumor group as those with average β-value differences of >0.3 and <−0.3 compared to NK, respectively. The numbers of selected hyper- and hypomethylated CpG probes in each tumor are listed in Table 2. Among them, the number of selected hypermethylated CpG probes mapped on the CpG island in CCSK, RTK, and ESFT were 490, 130, and 66, respectively, while those of hypomethylated non-CpG probes were 117, 320, and 136, respectively (Table 2).

**Table 2:** The number of hyper- and hypomethylated genes in comparison with non-neoplastic kidney.

	CpG island probe	
	Hypermethylated	Hypomethylated
CCSK	490 probes (437 genes)	46 probes (36 genes)
RTK	130 probes (107 genes)	65 probes (62 genes)
ESFT	66 probes (58 genes)	55 probes (46 genes)
	**Non-CpG island probe**	
	**Hypermethylated**	**Hypomethylated**
CCSK	184 probes (166 genes)	117probes (112 genes)
RTK	179 probes (160 genes)	320probes (275 genes)
ESFT	113 probes (101 genes)	136probes (124 genes)

Hypermethylation: difference of average β-value >0.3. Hypomethylation: difference of average β-value<−0.3.

**Figure 1:**
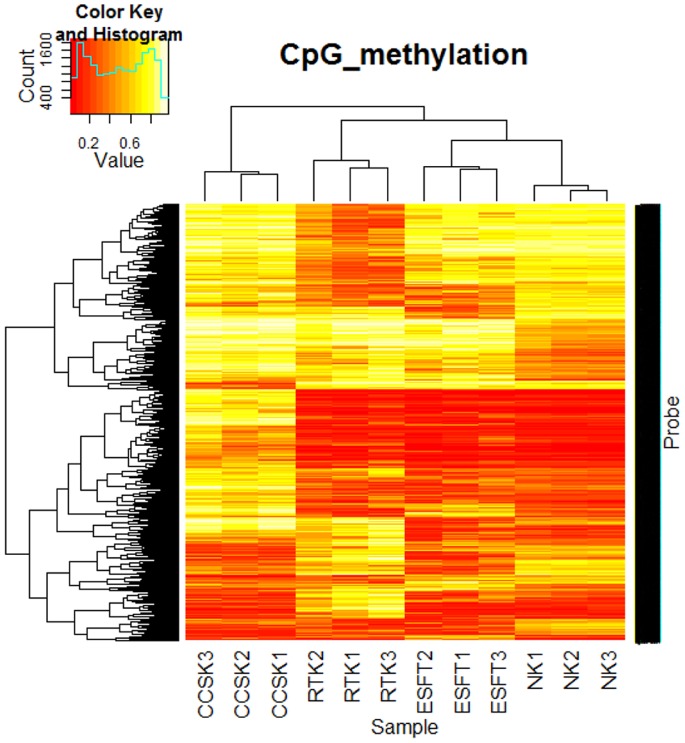
Hierarchical cluster analysis of methylation value (β) in Infinium assay on pediatric tumors. Two-way hierarchical cluster analysis of the methylation level of 1,494 probes (rows, equivalent to hyper- and hypomethylated genes shown in Table 2) and three cases for each of clear cell sarcoma of the kidney (CCSK), rhabdoid tumor of the kidney (RTK), the Ewing’s sarcoma family of tumors (ESFT), and non-neoplastic kidney (NK) (columns) were performed using hclust in the R clustering package. The β-values ranged from 0 (unmethylated) to 1 (fully methylated) on a continuous scale.

To test whether the tumors can be distinguished by the methylation level, we performed two-way hierarchical cluster analysis of methylation patterns using hyper- and hypomethylated sites (1,494 probes; equivalent to 1,281 genes selected in Table 2). As shown in Figure 1, each case in the same tumor was clustered in the same group, indicating the tumor-type-dependent methylation pattern of the selected probes.

To further select marker genes for methylation-based tumor-type classification, we defined tumor-specific differentially methylated genes as those with average β-value differences of >0.3 (hypermethylated) or <−0.3 (hypomethylated) compared to each of other tumor groups and NK. As shown in Table 3, 270 and 28 genes were identified as CCSK-specific hyper- and hypomethylated genes, respectively, while 65 and 155 genes were selected as RTK-specific hyper- and hypomethylated genes, respectively.

**Table 3:** The number of specifically methylated genes compared with other each of tumors and non-neoplastic kidney.

	Hypermethylation	Hypomethylation
CCSK	270 genes	28 genes
RTK	65 genes	155 genes
ESFT	7 genes	35 genes

Hypermethylation: difference of average β-value >0.3. Hypomethylation: difference of average β-value<−0.3.

Employing MassARRAY, next we analyzed the surrounding CpG of some specific probes filtered in Table 2, in detail to validate the results of an Infinium assay and further search for candidate genes for a differential diagnostic marker of renal sarcomas. Genes *ALDOC*, *CREG1, PKN1*, and *Thrombospondin-1* (*THBS1)* were selected because of their tendency to be hypermethylated in CCSK by the Infinium assay, while *ADRA1D* was selected because of distinct methylation levels among tumors. *MGMT* and *PTEN* were selected because of hypermethylation in 3 tumors, while *VHL* was selected because of hypomethylation in CCSK and RTK. These are known as tumor suppressor genes and these methylation changes are reportedly involved in specific tumors. [17], [18].

**Figure 2:**
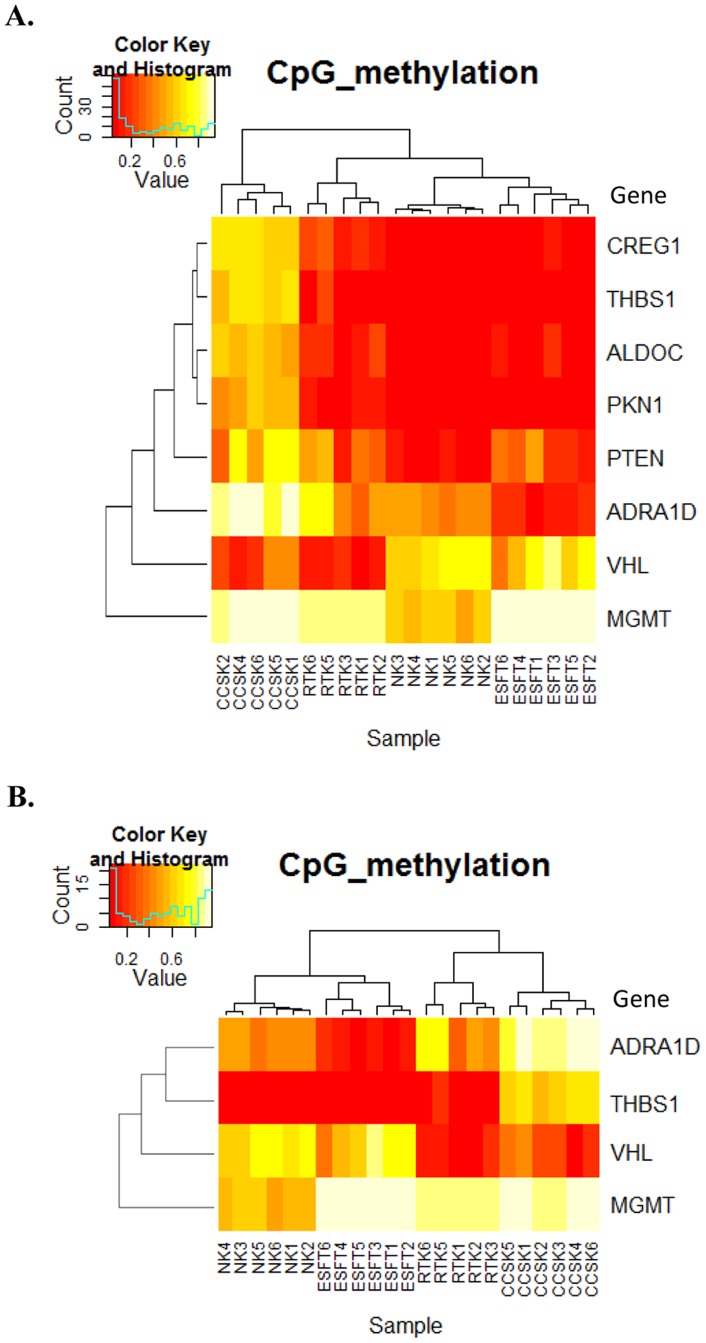
Hierarchical cluster analysis of methylation level of CpG analyzed by MassARRAY. (A) Based on the results indicated in Figure 1, 8 genes were selected as described in the text and analyzed by MassARRAY. Two-way hierarchical cluster analysis was performed using the CpG methylation average of all CpG that have passed QC from each gene. Two cases (RTK4 and CCSK3) showed failed analysis of some CpG sites, and were excluded from hierarchical analysis. (B) Four genes were further selected, and cluster analysis using the methylation average was performed as in (A). CCSK3 was successfully analyzed in all four genes, and included in this analysis.

We analyzed 6 each of RTK, CCSK, ESFT, and NK, the results of MassARRAY were well correlated with those of the Infinium assay, and each type of tumor revealed a specific DNA methylation pattern (Figure S2). In fact, when we performed two-way hierarchic cluster analysis using the CpG methylation average derived from the results, the cases for each tumor type were successfully classified into the same group (Figure 2A). As shown in Figure 2A, *CREG1, ALDOC, THBS1,* and *PKN1* were hypermethylated in CCSK, whereas RTK, ESFT, and NK were hypomethylated at specific CpG loci, as analyzed by the Infinium assay. *PTEN* was hypomethylated in NK, while variably methylated in sarcoma cases. *ADRA1D* was also hypermethylated in CCSK but hypomethylated in ESFT in comparison with NK, whereas variably methylated in RTK. Although *VHL* was hypomethylated in CCSK and RTK, it was hypermethylated in NK (Figure S3 and Figure S4). *MGMT* was hypermethylated in tumor groups compared to NK.

To distinguish these tumors by the DNA methylation pattern more simply, we further selected four genes characteristically methylated among different tumor groups: *ADRA1D, MGMT*, *VHL,* and *THBS1*, and performed cluster analysis using the methylation average of 4 genes. As shown in Figure 2B, CCSK, RTK, and ESFT were successfully classified according to the DNA methylation pattern of these genes, suggesting that the DNA methylation analysis of these 4 genes is sufficient for the differential diagnosis of these tumors.

**Figure 3:**
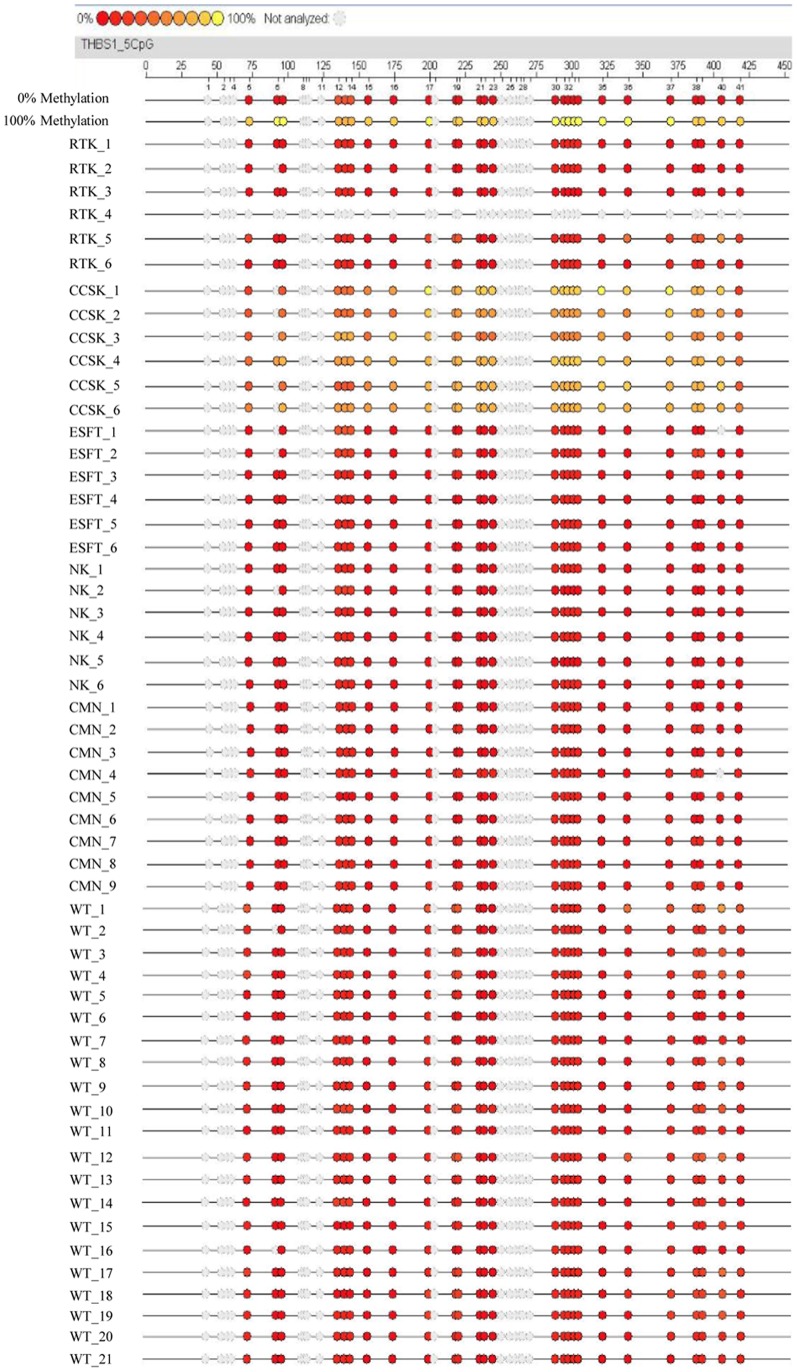
MassARRAY analysis of methylation in *THBS1*. In addition to the 6 cases for each tumor group, 9 and 21 cases of CMN and Wilms’ tumor (WT), respectively, were analyzed for DNA methylation of the *THBS1* CpG site, as in Figure 2A. Different colored of circles mark the position of CpG within the sequence (straight line) and the levels of methylation are shown in color (red, low methylation level; yellow, high methylation level). Gray circles represent the unanalyzed CpG sites. WT: Wilms’ tumor.

Since *THBS1* was found to be characteristically hypermethylated in CCSK (Figure 2B), we next examined whether the hypermethylation of *THBS1* alone can distinguish CCSK from other tumor groups. To confirm the specificity of *THBS1* hypermethylation in CCSK among pediatric renal tumors, we additionally analyzed Wilms’ tumor and CMN. As shown in Figure 3, when we analyzed 6 each of RTK, CCSK, ESFT, and NK as well as 21 cases of Wilms’ tumor and 9 cases of CMN, the CpG sites of *THBS1* were unmethylated in all of the cases, indicating that the CpG sites of *THBS1* are specifically hypermethylated in CCSK among pediatric renal tumors.

**Figure 4:**
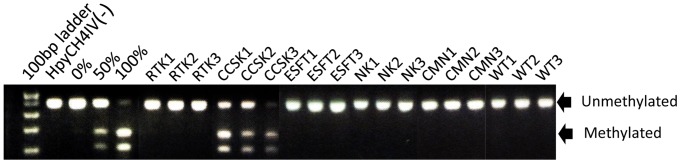
Combined bisulfite restriction analysis (COBRA) of *THBS1* in pediatric renal tumors. Using the same PCR products as in Figure 3, COBRA analysis was performed by digesting with the HpyCH4IV enzyme. The HpyCH4IV site is equivalent to CpG18 (chr15:37,660,642-37,660,645/hg18) in Figure 3. The positions of bands representing methylated and unmethylated DNA are indicated by arrows. As the control of HpyCH4IV digestion, 0, 50, and 100% methylated DNA were loaded on the same gel. WT: Wilms’ tumor.

To detect the methylation of *THBS1* more easily, we have developed combined bisulfite restriction analysis (COBRA) and analyzed 21 cases of CCSK and 41 cases of Wilms’ tumor, 6 cases of RTK, 9 cases of CMN, and 6 cases of NK. As shown in Figure 4 and Table 4, the digestion of bisulfite PCR products with HpyCH4IV clearly indicated that a CpG site of *THBS1* in all CCSK cases was hypermethylated. However, none of other tumor groups exhibited hypermethylation of the CpG site of *THBS1.* The results strongly indicate that hypermethylation of *THBS1* is a specific characteristic of CCSK among pediatric renal tumors, and could be utilized as diagnostic maker of this tumor.

**Table 4:** Frequency of *THBS1* methylation by COBRA.

	Hypermethylated cases
RTK	0/6
CCSK	21/21
ESFT	0/6
CMN	0/6
Wilms	0/41
NK	0/6

## Discussion

In this study, we carried out a DNA methylation analysis to identify genes differentially methylated in a series of pediatric tumors and we clearly showed that different pediatric sarcomas occurring in the kidney possess a distinct DNA methylation profile. Especially, CCSK is more frequently hypermethylated, but less hypomethylated, at the CpG island, compared with other tumors. Since pediatric renal sarcomas have overlapping morphologic and clinical features, the significant differences in epigenetic characteristics between these tumors are of particular interest and may represent the distinction of their cell origin or developmental mechanism. We also showed that these tumors can definitely be classified based on the DNA methylation profile, indicating the usefulness of epigenetic profiling for the differential diagnosis of pediatric renal sarcomas, and found that a combination of four genes is sufficient. Furthermore, the DNA methylation status of the *THBS1* CpG site detected by COBRA alone can distinguish CCSK cases from other pediatric renal tumors, including Wilms’ tumor and CMN.

The pathological diagnoses of pediatric renal tumors are often supported by immunohistochemical and molecular genetic findings. For example, biallelic inactivation of *SMARCB1* as evidenced by negative immunohistochemical staining has high sensitivity and specificity for the diagnosis of RTK, and *ETV6-NTRK3* fusion is a marker for CMN of the cellular type. In the case of CCSK, however, it has neither immunohistochemical nor molecular genetic markers, while *YWHAE-FAM22* fusion was reported in only a minority [19]. Since *THBS1* hypermethylation is highly specific for CCSK, as we presented in this study, this finding should be useful for a molecular marker of this tumor. Especially, COBRA of the *THBS1* CpG site is highly accurate, reproducible, and can be performed without particular equipment, and, thus, it could be a candidate for routine examination for the differential diagnosis of CCSK from other pediatric renal tumors.

DNA methylation has been proposed as a diagnostic marker for certain cancers. For example, Goto reported that malignant mesothelioma could be differentiated from lung adenocarcinoma by methylation profiles [20], and Mahoney reported that embryonal and alveolar subtypes of rhabdomyosarcoma have a distinct DNA methylation profile [21]. In their cases, however, the methylation status of at least several genes was required for the differentiation of only two entities. In contrast, our findings showed that hypermethylation of a single locus of *THBS1* is sufficient for the differentiation of CCSK from other pediatric tumors and it could serve as a robust diagnostic marker for this tumor.

Hypermethylation of the *THBS1* CpG site has been observed in some tumors. For example, Guo et al. reported that the rate of methylation of *THBS1* was significantly higher in gastric cardia adenocarcinoma than that in the corresponding normal tissues and accompanied by reduction of its mRNA and protein expressions [22], and Guerrero et al. also reported that the hypermethylation of *THBS1* is associated with a poor prognosis in penile squamous cell carcinoma [23]. In both cases, the hypermethylation of *THBS1* has been suggested to be correlated with the pathogenesis.

THBS1 is a member of the thrombospondin family, and is known for putative antiangiogenic factor [24], [25]. By employing a knockout mouse model, several studies have shown that the absence of *THBS1* leads to increased vascularization and THBS1 protein inhibits tumor progression in several ways, including direct effects on cellular growth and apoptosis in the stromal compartment [26]–[29]. Since CCSK is known to be rich in a fine vascular network, it is reasonable to presume that hypermethylation of the *THBS1* CpG site is involved in the pathogenesis of CCSK. However, we observed no significant correlations between the methylation status and expression of THBS1 by realtime RT-PCR and immunohistochemistry (data not shown). This is possibly due to hypermethylated CpG sites of *THBS1* in CCSK that we identified as not being responsible for *THBS1* expression, and other CpG sites are related to the regulation of *THBS1* expression. In fact, other CpG probes (cg19570574: chr15: 37660116–37660117/hg18, cg04051458: chr15: 37660352–37660353/hg18) in the upstream region of the *THBS1* transcription start site were hypomethylated in CCSK based on our assay. Another possibility is that THBS1 is expressed in a limited period during tumorigenesis. Further studies to elucidate the biological significance of CCSK hypermethylation are now underway.

In conclusion, pediatric renal sarcomas possess a distinct DNA methylation profile in a tumor-type-specific manner. The DNA methylation status of the *THBS1* CpG site detected by COBRA alone is sufficient for the distinction of CCSK from other pediatric renal tumors. Although further analysis to elucidate the biological importance of the differential DNA methylation of each tumor is required, our observation should shed light on the significance of the epigenetic diversity of pediatric renal tumors on their biological features and mechanism of pathogenesis.

## Supporting Information

Figure S1 **Images of frozen tissue sample by H.E. staining.** Frozen tumor tissues were embedded in OCT-compound and sectioned in 6 µm, stained with H.E. The proportion of viable tumor tissue was evaluated under light microscope.
(TIF)

Figure S2 **Correlation of the methylation values between Infinium assay and MassARRAY.** In cases of *THBS1* and *CREG1*, methylation values of same CpG site measured by MassARRAY (average of 6 samples) and Infinium BeadChip Assays (average of 3 samples) were indicated in the scattergrams and values of coefficient of determination (R^2^) were calculated. In case of *VHL*, two probe sites were shown. Since each site of *VHL* could not be discriminated from the neighbouring site by MassARRAY, the methylation value was obtained as an average of two sites. Coefficient of determination between the values obtained by two methods was larger than 0.99 in each case. Methylation levels of the equivalent CpG sites were correlated between Infinium assay and MassARRAY.
(TIF)

Figure S3 **MassARRAY analysis of** ***VHL*** **in pediatric renal tumors.** MassARRAY analysis of the *VHL* was carried out in 6 each of RTK, CCSK, ESFT, NK. Different colored of circles mark the position of CpG within the sequence (straight line) and the levels of methylation are shown in color (red, low methylation level; yellow, high methylation level). Gray circles represent the unanalyzed CpG sites. CpG5 and CpG13 are equivalent to Infinium assay probes.
(TIF)

Figure S4 **Combined bisulfite restriction analysis** (**COBRA) of** ***VHL*** **in pediatric renal tumors.** Bisulfite PCR amplification of *VHL* was carried out by using same primer for MassArray. COBRA analysis was performed by digesting with the TaqI restriction enzyme. The TaqI site is equivalent to CpG3 in MassARRAY analysis. The site of genome sequence is CCGA, which is converted to TaqI sequence (tCGA) by bisulfite reaction. PCR amplification of RTK4 was failed. The digested DNA was separated on 2% agarose gels in 1×TAE buffer, stained with ethidium bromide, and visualized on a UV transilluminator.
(TIF)

Table S1 Numbers of probes filtered with p-value and SD.
(DOCX)
