# Supplementary figures and images for: DNA Methylation Profile Distinguishes Clear Cell Sarcoma of the Kidney from Other Pediatric Renal Tumors
Source: PLoS One. 2013 Apr 26;8(4):e62233. doi: 10.1371/journal.pone.0062233 (PMC3637380; doi:10.1371/journal.pone.0062233)

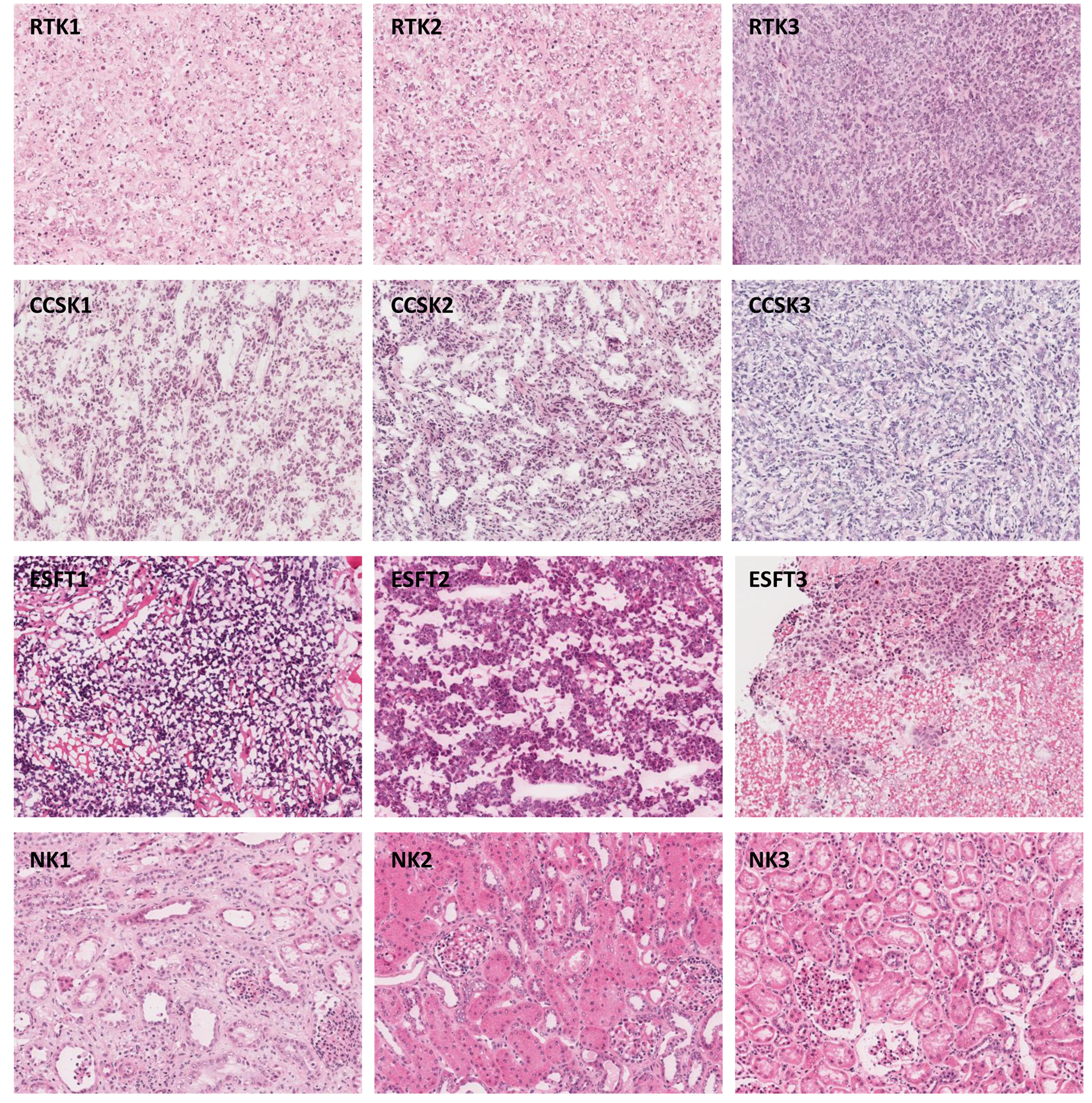

Supplement: Figure S1 — Images of frozen tissue sample by H.E. staining. Frozen tumor tissues were embedded in OCT-compound and sectioned in 6 µm, stained with H.E. The proportion of viable tumor tissue was evaluated under light microscope. (TIF) [file pone.0062233.s001.tif]

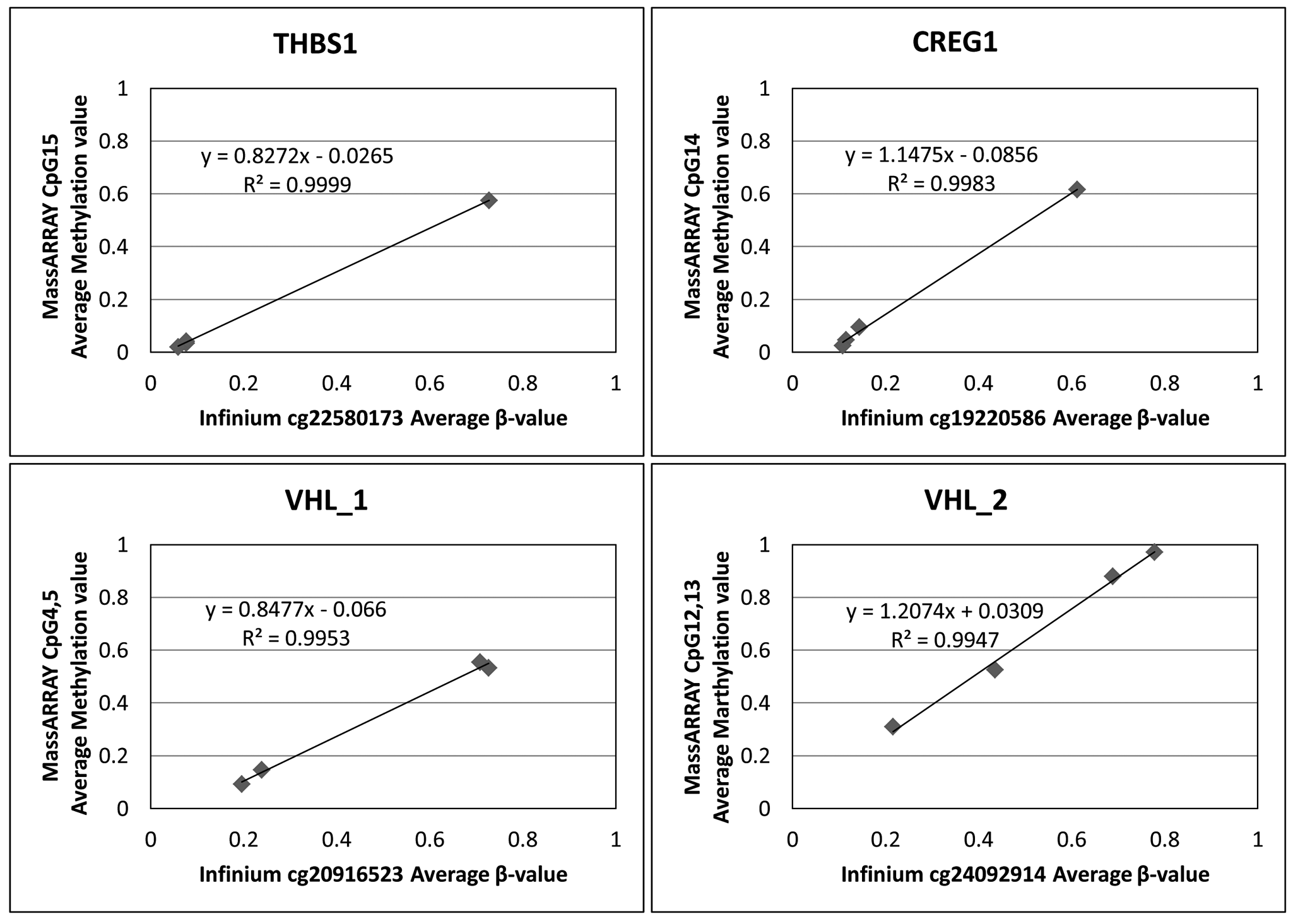

Supplement: Figure S2 — Correlation of the methylation values between Infinium assay and MassARRAY. In cases of THBS1 and CREG1, methylation values of same CpG site measured by MassARRAY (average of 6 samples) and Infinium BeadChip Assays (average of 3 samples) were indicated in the scattergrams and values of coefficient of determination (R2) were calculated. In case of VHL, two probe sites were shown. Since each site of VHL could not be discriminated from the neighbouring site by MassARRAY, the methylation value was obtained as an average of two sites. Coefficient of determination between the values obtained by two methods was larger than 0.99 in each case. Methylation levels of the equivalent CpG sites were correlated between Infinium assay and MassARRAY. (TIF) [file pone.0062233.s002.tif]

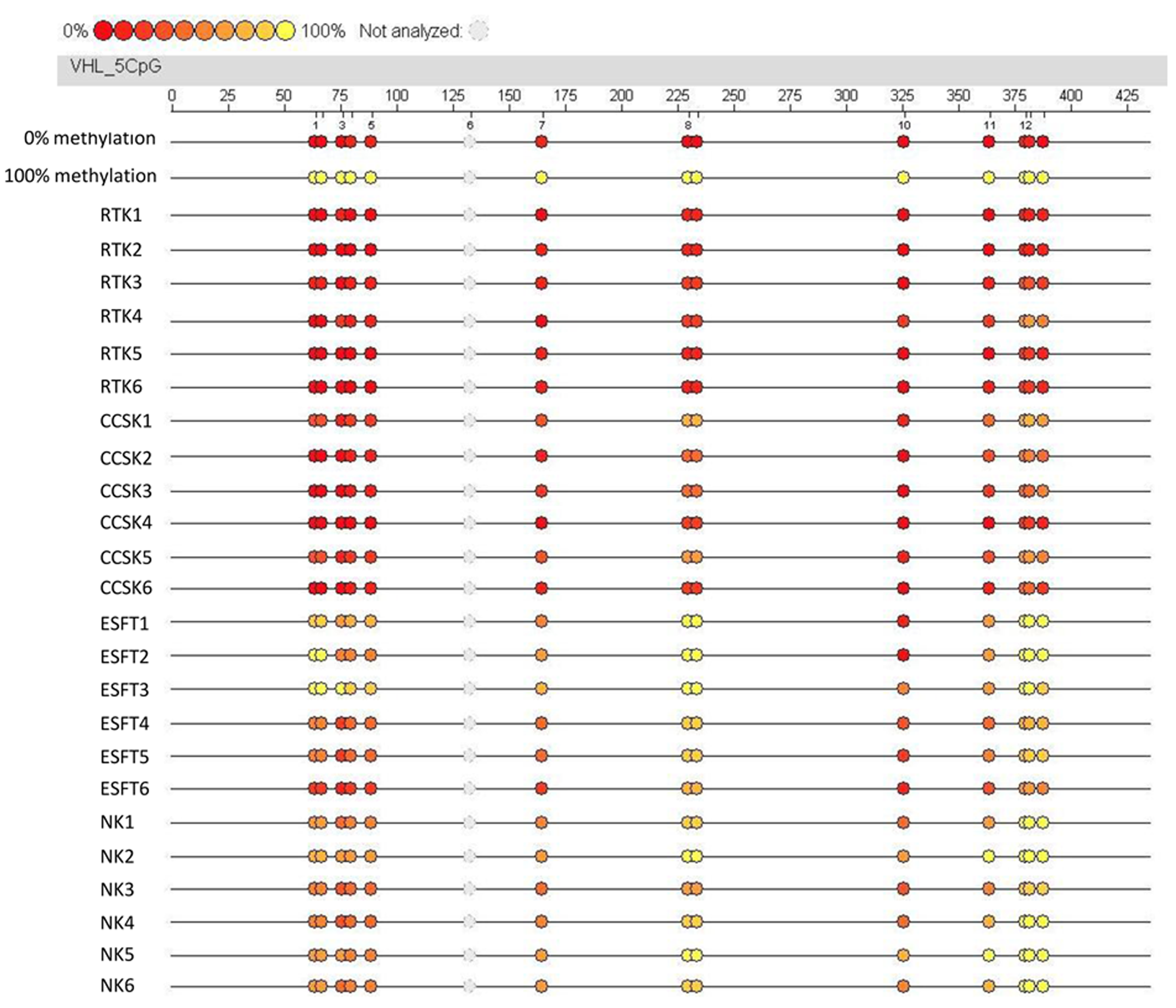

Supplement: Figure S3 — MassARRAY analysis of VHL in pediatric renal tumors. MassARRAY analysis of the VHL was carried out in 6 each of RTK, CCSK, ESFT, NK. Different colored of circles mark the position of CpG within the sequence (straight line) and the levels of methylation are shown in color (red, low methylation level; yellow, high methylation level). Gray circles represent the unanalyzed CpG sites. CpG5 and CpG13 are equivalent to Infinium assay probes. (TIF) [file pone.0062233.s003.tif]

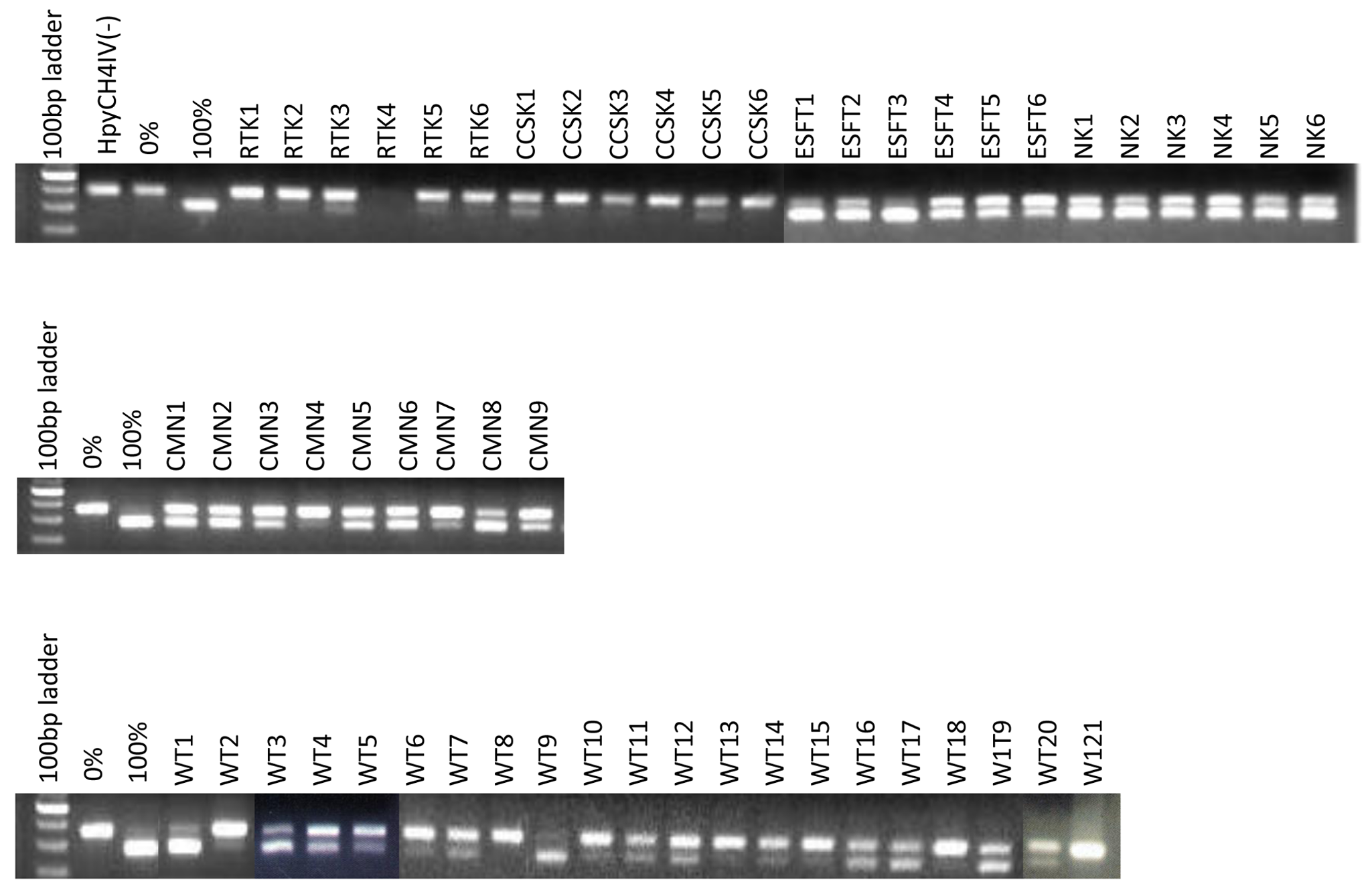

Supplement: Figure S4 — Combined bisulfite restriction analysis (COBRA) of VHL in pediatric renal tumors. Bisulfite PCR amplification of VHL was carried out by using same primer for MassArray. COBRA analysis was performed by digesting with the TaqI restriction enzyme. The TaqI site is equivalent to CpG3 in MassARRAY analysis. The site of genome sequence is CCGA, which is converted to TaqI sequence (tCGA) by bisulfite reaction. PCR amplification of RTK4 was failed. The digested DNA was separated on 2% agarose gels in 1×TAE buffer, stained with ethidium bromide, and visualized on a UV transilluminator. (TIF) [file pone.0062233.s004.tif]
