# Supplementary material for: DNA Methylation Profile Distinguishes Clear Cell Sarcoma of the Kidney from Other Pediatric Renal Tumors
Source: PLoS One. 2013 Apr 26;8(4):e62233. doi: 10.1371/journal.pone.0062233 (PMC3637380; doi:10.1371/journal.pone.0062233)
Supplement: Table S1 — Numbers of probes filtered with p-value and SD. (DOCX) [file pone.0062233.s005.docx]

**Table S1. Numbers of probes filtered with p-value and SD.**

|  | Number of detected probes | | | |
| --- | --- | --- | --- | --- |
|  | Detection p-value<0.05 | Detection p-value<0.05 for all samples | Detection p<0.05 for all samples and SD<0.2 within each entity | Detection p-value<0.05 for all samples and SD<0.2 within each entity for all 4 entities |
| ESFT1 | 27529 | 27400 | 25877 | 23700 |
| ESFT2 | 27501 |  |  |  |
| ESFT3 | 27530 |  |  |  |
| RTK1 | 27519 |  | 25256 |  |
| RTK2 | 27507 |  |  |  |
| RTK3 | 27527 |  |  |  |
| CCSK1 | 27548 |  | 26734 |  |
| CCSK2 | 27432 |  |  |  |
| CCSK3 | 27570 |  |  |  |
| NK1 | 27552 |  | 27387 |  |
| NK2 | 27528 |  |  |  |
| NK3 | 27521 |  |  |  |
